# Pharmacy Students’ Perceptions of the Pharmacist Role: An Arts-Informed Approach to Professional Identity Formation

**DOI:** 10.3390/pharmacy11050136

**Published:** 2023-08-30

**Authors:** Meghan Noyen, Ravina Sanghera, Janice Y. Kung, Theresa J. Schindel

**Affiliations:** 1 College of Health Sciences, Faculty of Pharmacy and Pharmaceutical Sciences, University of Alberta, Edmonton, AB T6G 2H7, Canada; 2 Office of the Dean of Students, University of Alberta, Edmonton, AB T6G 2H7, Canada; 3 John W. Scott Health Sciences Library, University of Alberta, Edmonton, AB T6G 2R7, Canada

**Keywords:** pharmacist role, professional identity, professional identity formation, pharmacy education, qualitative research, arts-informed research, draw-and-write technique, discourse analysis

## Abstract

Elements of professional identity are closely intertwined with professional roles, and individuals perceive themselves in relation to their roles. How pharmacists perceive their professional identity influences how they enact their roles in practice. For pharmacy students, understanding the pharmacist role and envisioning themselves in that role supports the formation of their professional identity. This study explores students’ perceptions of the pharmacist role. First-year pharmacy students enrolled in the Doctor of Pharmacy program at the University of Alberta were invited to participate in this study. Using an adapted version of the draw-and-write technique, participants were asked to express their understanding of the pharmacist role visually. An analysis of the results was guided by established discourses related to pharmacist identity derived from pharmacy education literature. In total, 100 pharmacy students participated in this study. The findings indicate that pharmacy students have a comprehensive understanding of the pharmacist role, especially the dispenser and health care provider aspects of a pharmacist’s professional identity. Additionally, students acknowledged the involvement of pharmacists in health care teams, in public health, and primary health care services. A discourse related to professional identity, the multi-faceted professional, emerged to describe the coexistence of multiple roles in modern pharmacy practice. An arts-based activity successfully facilitated the exploration of pharmacy students’ perceptions of the professional role of pharmacists. This approach has potential in supporting instruction regarding professional identity formation within the curriculum.

## 1. Introduction

The concept of professional identity has garnered significant attention in recent years from researchers investigating various professions, including pharmacy. Educators now prioritize the socialization of pharmacy students into the profession to facilitate formation of their professional identity as they transition to practicing pharmacy [1,2,3,4,5]. This support of professional identity formation is particularly crucial given the dynamic nature of the pharmacy profession and the significant changes in the pharmacist’s role within society over the last century [6]. Students with a comprehensive understanding of this role are well positioned to engage in lifelong learning, adapt their practice, navigate crises, and exert influence within the pharmacy profession and health care system [3,5,7,8,9].

The term “professional identity” refers to how professionals define themselves in relation to their professional roles, encompassing both their sense of identity and the expected behaviors associated with their roles [10,11,12]. How professionals perceive their roles shapes their understanding and influences how they enact those roles in work situations [12,13]. Two central questions that relate to professional identity are “who am I?” and “what do I do?” [14].

Professional identity is constructed continuously throughout people’s careers [15]. This process is influenced by individual and institutional factors [11,16], changes in work [16,17], prevailing societal discourses about professional roles [18], alignment between personal values and work [12], interactions with fellow professionals [11,12,19], involvement in professional organizations [20,21], and past experiences [13]. Professional identity is therefore a fluid, evolving, and socially constructed phenomenon [10].

Professional identity formation is a complex and iterative process of socialization that occurs gradually over time, a process whereby individuals learn to “think, act, and feel” like professionals [22]. Within pharmacy education, professional identity formation is considered a major curricular goal [1,3,23]. To this end, educational programs may include elements such as acquiring knowledge in the field, engaging in reflective writing, interacting with peers, observing role models, receiving feedback, and benefiting from mentoring relationships [5,24,25,26,27,28,29,30]. Arts-based activities, including drawing, writing, mask-making, and storytelling, have been employed to support the formation of professional identity for medical students [31,32,33,34,35]. These activities have not, however, been widely utilized in programs with pharmacy students, despite the fact that pharmacy educators are encouraged to bring creativity, curiosity, adaptability, and responsiveness into the curriculum to support professional identity formation [5].

The professional role of the pharmacist includes social titles such as “apothecary” or “pharmacist”, both of which are closely linked to professional identity; various similar titles may be integrated into the self-concept of pharmacy practitioners [36]. In pharmacy education, early exposure to and exploration of the pharmacist role in society is important to professional identity formation [1,2,5,26]. Students’ perceptions of this role are integral to the process of professional identity formation [37]. Previous research has suggested that prior to starting their pharmacy education, most students have limited awareness of the role of pharmacists in society [1,38,39] and a limited understanding of pharmacy practice [7,40]. Gaining a clear understanding of students’ perceptions of the pharmacist role at the start of their formal education will assist educators in designing effective learning activities to support the development of professional identity. The aim of this study is to explore how students entering the Doctor of Pharmacy program perceive the role of pharmacists.

## 2. Materials and Methods

### 2.1. Qualitative Study Design

This qualitative study draws upon social constructionism and arts-informed research. A social constructionist perspective emphasizes discourse as the means through which individuals articulate their understanding of the self and the world [18]. Central to this perspective is the recognition that language plays a vital role in the process of meaning construction. Discourses encompass a collection of meanings, representations, images, stories, statements, and other elements that collectively shape a specific interpretation of events [18]. Texts, including words and drawings, are concrete representations of discourses. Language, both written and spoken, impacts what individuals actually do and what they are permitted to do in society.

Arts-informed research is a type of qualitative research influenced by the arts [41]. It deepens our comprehension of human experience by utilizing a non-traditional approach and representational methods of inquiry, broadening the reach of scholarly work by increasing accessibility to diverse audiences. For example, qualitative research methods involving drawings have been used to examine how individuals understand their experiences in the world [42,43]. Engaging in arts-based activities allows study participants to express ideas and experiences that may be difficult to convey solely through words [41,43]. Researchers can also observe and communicate about a diverse range of experiences using art. Drawings have been utilized in pharmacy education research to explore the experiences of pharmacy students [44] and educators [45], and to clarify pharmacy students’ beliefs about pharmacists’ role and practices [46]. The design of this study recognizes that language and drawings are crucial in understanding, shaping, and limiting the ways individuals feel, think, and act as pharmacists.

### 2.2. Context

The Faculty of Pharmacy and Pharmaceutical Sciences, College of Health Sciences at the University of Alberta offers an entry-to-practice Doctor of Pharmacy (PharmD) degree program [47]. Students complete a minimum of two years of prerequisite courses before commencing the four-year program. As part of the application process, students are required to submit a letter outlining their career goals, knowledge of the profession, and related experience. They must also consult with a pharmacist to learn about the profession [48]. The program is divided into three terms each year: fall, winter, and spring/summer. It encompasses foundational classroom, laboratory, and practice-based courses across six curricular streams including pharmaceutical sciences, administrative and social pharmacy, pharmacotherapy, patient care skills, interprofessional, and practical experience. Students join the program in the fall term commencing in September with mandatory in-person orientation sessions held throughout the first several weeks of the term. (These orientation sessions were delivered in a hybrid format in fall 2021, as not all students had yet returned to in-person education.) At the time of data collection for this study, students attended one lecture that introduced the role of the pharmacist.

In the province of Alberta, Canada, pharmacy practice is wide in scope and includes accessing patient information through provincial electronic health records, ordering laboratory tests, administering medications via injection, and prescribing [49]. All pharmacists are authorized to prescribe in emergency situations, adapt prescriptions from other prescribers, and authorize medication refills. Pharmacists with additional prescribing authorization can also initiate new medication therapy [50]. During the COVID-19 pandemic, community pharmacists were authorized to renew prescriptions for narcotic and controlled drugs [51]. The provincial plan provides compensation to community pharmacies for some patient care services provided by pharmacists, such as medication assessments, care plan development, and vaccinations [52].

### 2.3. Recruitment

Year 1 pharmacy students entering the PharmD program in the fall of 2021 were eligible to participate in the study. Participants were recruited via email invitation from the Student Services office at the Faculty of Pharmacy and Pharmaceutical Sciences. Recruitment and study materials were distributed to students in an envelope as part of the welcome package that students received prior to the session.

### 2.4. Research Team and Reflexivity

The research team comprised four female members: two pharmacist researchers (RS, TJS), one health librarian (JYK), and one pharmacy student (MN) in the third year of the PharmD program. One researcher (TJS) had established research credentials, while both RS and TJS had extensive experience in pharmacy practice. Additionally, MN, the pharmacy student, brought an artistic perspective to the team. One member (TJS) had prior experience utilizing the draw-and-write technique. All members of the research team shared a belief that the formation of professional identity begins early in the academic journey, potentially before students officially begin their program. They wanted to know the extent of new pharmacy students’ understanding of the role of pharmacists in society. All team members anticipated that the draw-and-write technique would yield valuable data and insights derived from students’ prior experiences and observations of pharmacists. In addition, all team members understood that their subjectivity could impact the results of the study. The reporting of this qualitative research adhered to the COREQ criteria [53].

### 2.5. Data Collection

Data were collected using an adapted version of the draw-and-write technique [54]. This technique is an arts-informed research method that has gained considerable popularity since the 1980s and was initially used in research with children [55]. It provides a creative avenue for investigating perceptions and understanding of experience within the world. Research participants are invited to create drawings to express their thoughts, after which they write or verbalize about their artwork. This technique is often supplemented by individual or focus-group interviews [55]. The draw-and-write technique has successfully been employed with adult participants [56,57,58]. Reflection has been incorporated [57] and a standardized data collection process, such as the iSquare protocol, may be utilized, as in this study [54,56].

**Figure 1:**
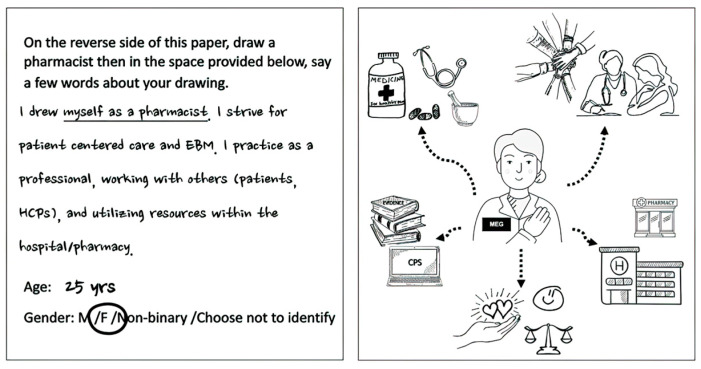
Sample pSquare.

Students were provided with a square piece of art paper (4.25 × 4.25”), referred to as pSquare, and a high-quality black ink gel pen. The pSquare was blank on one side. Students were verbally instructed to draw a response to the question, “What is the pharmacist’s role?”, after which they wrote a few words about their drawings in the space provided on the other side of the paper. Two optional questions asked about demographic data (age and gender). The activity required 7 min to complete, after which students placed the pSquare in an envelope. In-person participants placed their envelopes in a collection box in the classroom. Remote participants were invited to drop off their envelopes at a designated drop-off spot (the Faculty of Pharmacy and Pharmaceutical Sciences Student Services office) the following week. One reminder email was sent regarding the opportunity to drop off completed pSquares. (See Figure 1 for an example of a completed pSquare.)

### 2.6. Data Analysis

All pSquares were scanned using an HP Officejet Pro 8610 scanner. The scanned images were manually stored in a secure folder shared among the research team members. Each image was given a designated number in the format of 2025-Y1-XXX and subsequently transferred to a PowerPoint slide. Each slide included the drawing, accompanying text about the drawing, and information about participants’ gender and age.

The drawings and associated text were analyzed using a discourse analysis approach [18,59]. This approach was chosen because of the central role of language in constructing meaning and influencing actions, as per the social constructionist perspective. The analysis was iterative and conducted in phases. In the first phase, one team member (MN) examined the drawings for their overall composition, artistic expression of the pharmacist role, and their alignment with two questions central to professional identity: 1) “Who am I?” and 2) “What do I do?” [14]. Compositional elements, such as contextual depiction, physical spacing, and size, were also observed and documented. The team convened, along with a local artist (Ruth-Anne French), to review the analytical approach applied to the drawings in the first 10 pSquares and to discuss preliminary coding, for example, care provider (who am I) and administer vaccines (what do I do). The second phase of the analysis was guided by the previously mentioned established identity discourses based on the work of Kellar and colleagues [60]. Analysis and coding was conducted independently by two team members (MN, TJS) with attention to identity discourse, image descriptions, symbols, and language [60]. These two team members reached consensus on the coding, categorization, and interpretation of the data. In the final phase of analysis, all research team members contributed to interpretation, synthesis, and categorization of data not represented by existing identity discourses.

### 2.7. Rigor

Several measures were taken to ensure trustworthiness of the study results, including: (1) documentation of the study context, participants, and process of interpreting the data, thereby providing a rich contextual understanding; (2) investigator triangulation, where two researchers independently conducted the analysis and engaged afterward in thorough discussions to validate interpretations; (3) theoretical triangulation involving an artist with expertise outside the main study area; (4) reflexivity during in-depth discussions of the researchers’ positions, reactions, and interpretations; (5) careful documentation at every stage of the research process; and (6) well-documented adaptation of the draw-and-write technique to prioritize participants’ perspectives throughout the research process [54,61].

The University of Alberta Research Ethics Board 1 approved this study (Pro00095356).

## 3. Results

In total, 100 pharmacy student participants submitted pSquares following a mandatory seminar as part of their orientation to year 1 of the Doctor of Pharmacy program on 14 September 2021. None of the students who attended the session remotely submitted drawings for the study. Participants were 19 to 40 years of age (Table 1). Overall, students in the class of 2025 (*n* = 124) resided in the province of Alberta (92%), had been previously employed in a health care field (41%), and had experience working in a pharmacy (27%).

We now present the results of the analysis organized according to identity discourses provided in the literature, as follows: apothecary, dispenser, merchandiser, expert advisor, and health care provider [60]. A sixth discourse emerging from this study, the multi-faceted professional, represents the combination of multiple aspects of identity in the work of pharmacists. Representative pSquare drawings and written text are available at https://doi.org/10.7939/r3-xyta-5g55 (accessed on 25 August 2023).

### 3.1. Apothecary

The apothecary identity discourse is associated with the practice of making medicines, encompassing both the art and science of the pharmacist role [60]. This aspect of identity combines elements of both the medical and pharmacy professions, including preparing medicines and providing medical care. A symbol linked to this aspect of identity is the mortar and pestle [60].

**Figure 2:**
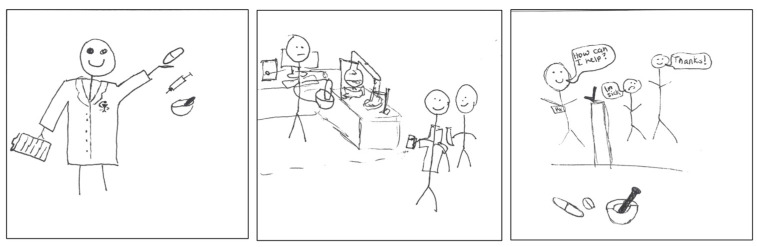
pSquares depicting the mortar and pestle symbol associated with the apothecary aspect of identity (2025-Y1-006, 2025-Y1-047, 2025-Y1-061 with text “How can I help? I’m sick. Thanks!”).

The mortar and pestle symbol appeared in participants’ drawings (Figure 2) and was associated with dispensing medications, compounding, administering medications by injection, and providing care to patients.

In the context of modern pharmacy practice, participants acknowledged the pharmacist role as having roots in science and medicine and described them as providing accessible care for people in the community:

“*[A] pharmacist is someone who is professional. Always in a lab coat, almost like a scientist. Always ready to help someone with questions [and] to serve the community.*”

[2025-Y1-035].

“*A pharmacist should be passionate to help. I believe that pharmacists, as [the] most accessible part of healthcare, are responsible to share their medical knowledge in [the] best way possible to help those in need and that comes with the love for medicine, pharmacy, and society.*”

[2025-Y1-070].

### 3.2. Dispenser

The dispenser identity discourse is primarily associated with the act of dispensing medications, though it also includes compounding and providing health-related information [60]. A symbol of this aspect of identity is the corner drug store [60].

**Figure 3:**
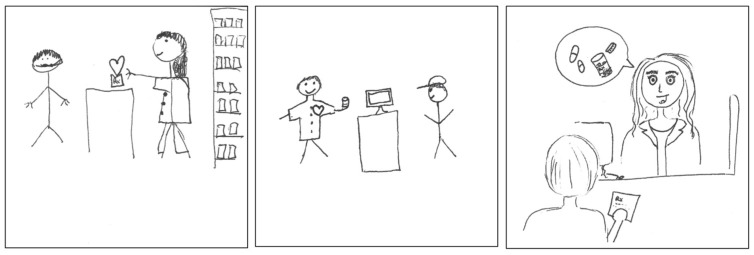
pSquares depicting the dispensing aspect of the pharmacist role associated with the dispenser identity (2025-Y1-018, 2025-Y1-059, 2025-Y1-089).

Participants emphasized dispensing as part of the pharmacist role, primarily within a community pharmacy environment, as depicted in their drawings (Figure 3). Illustrations associated with the dispenser identity discourse were found in more than 40% of the pSquares in the dataset, many of which also featured drawings of buildings (the corner drug store symbol) associated with the dispenser aspect of identity [2025-Y1-001], in addition to pharmacy counters [2025-Y1-021] and medication shelves [2025-Y1-024]. Some of the drawings conveyed the complexity of the dispensing aspect of the pharmacist role, indicating a recognition that it involves more than simply dispensing medications. The text accompanying the first pSquare below (Figure 3) stated:

“*Pharmacists have a significant role in patient care beyond just dispensing medications.*”

[2025-Y1-018].

Other symbols and material objects are depicted, including caring (hearts), providing medication therapy (vials, tablets, capsules, prescriptions), collecting patient information (computer screens), counseling (text bubbles), and providing clinical services (clipboard). Participants described the dispensing aspect of the pharmacist role as follows:

“*I drew a pharmacist next to a vial and computer. The vial represents the pharmacist’s medication knowledge. The computer represents patient information collected by a pharmacist.*”

[2025-Y1-003].

“*White coat = professional, pill = dispensing medication, syringe = admin[istration] of injections, mortar & pestle = compounding, clipboard = clinical aspect.*”

[2025-Y1-006].

**Figure 4:**
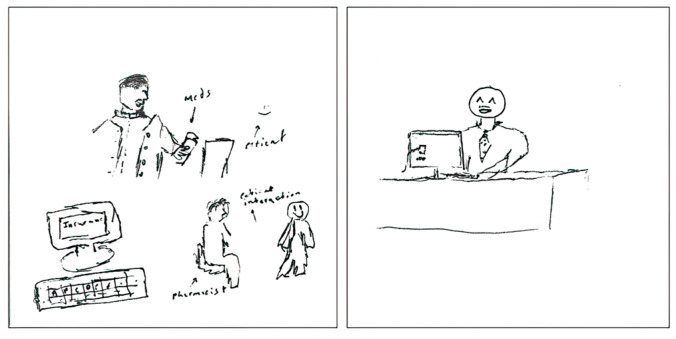
pSquares depicting the information role (2025-Y1-030 with text “Insurance. Meds. Patient. Pharmacist. Patient interaction.”, 2025-Y1-087).

The dispenser identity discourse was also represented by illustrations of pharmacists providing health information in conjunction with dispensing and counseling activities, as well as in a standalone service context, as in Figure 4. The accessibility of pharmacists as health care professionals was emphasized in the text accompanying the drawing in the second pSquare:

“*The pharmacist is a professional, but welcoming face for people to interact with. It is important for patients to have pharmacists to ask them for medical information.*”

[2025-Y1-087].

### 3.3. Merchandiser

The merchandiser aspect of identity is linked to the commercialization of the pharmacy profession [60]. It is often perceived as unfavorable due to the inherent conflict between prioritizing public service and complying with corporate agendas. The symbol closely associated with the merchandiser identity discourse is the villain, who is often depicted accompanied by objects that symbolize corporate management and ownership, business practices, and profit-seeking [60].

**Figure 5:**
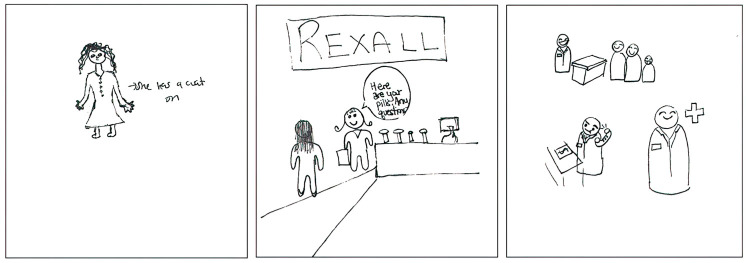
pSquares depicting the merchandiser aspect of identity (2025-Y1-050 with text “She has a coat on”, 2025-Y1-053 with text “REXALL. Here are your pills. Any questions?”, 2025-Y1-100).

However, the representation of the merchandiser aspect of identity in the dataset was limited. The merchandiser aspect of identity was observed in three specific pSquares, as illustrated in Figure 5. The first pSquare depicts a pharmacist in an ownership role, described in the written text as follows:

“*My pharmacist that I drew is a female and she owns her own pharmacy. She’s very polite and always does her best for patients.*”

[2025-Y1-050].

The second drawing incorporates a reference to a specific corporate pharmacy chain, Rexall [2025-Y1-053], while the third pSquare includes a dollar symbol in association with a slogan:

“*Serve your community, resolve problems, represent and advance health care.*”

[2025-Y1-100].

It is important to note that none of the pSquares contained undesirable or villainous portrayals of pharmacists.

### 3.4. Expert Advisor

The expert advisor aspect of identity is associated with the pharmacist as a consultant or advisor to physicians [60]. This identity discourse acknowledges pharmacists as experts and knowledgeable professionals in their field. It is symbolized by the image of a pharmacist providing advice or guidance [60].

**Figure 6:**
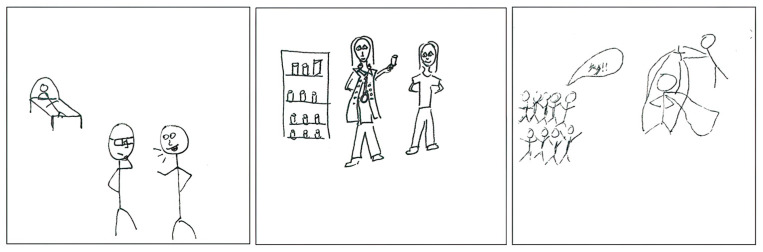
pSquares depicting the expert advisor identity discourse (2025-Y1-033, 2025-Y1-086, 2025-Y1-039 with text “Yay!!”).

The pharmacist as an expert advisor to physicians or other health care providers was observed in the dataset, as depicted in Figure 6. The first pSquare portrays a pharmacist engaging in discussions and offering input on treatment options with a physician in a hospital setting, acting as an advisor:

“*A doctor and a pharmacist are shown discussing potential treatment options. The pharmacist is providing their input.*”

[2025-Y1-033].

The written text accompanying the drawing in the second pSquare emphasizes the pharmacist as advisor to other health care providers:

“*Pharmacists have a role in providing expertise to patients and other health care providers in the realm of pharmaceutics and pharmacotherapy. Their ultimate goal is to enhance patients’ well-being.*”

[2025-Y1-086].

The third pSquare draws attention to the (often undervalued) expertise of the pharmacist and the behind-the-scenes nature of the work of the expert advisor:

“*The drawing illustrates people applauding a health care professional (e.g., physician, nurse), while the pharmacist is depicted as pulling strings, symbolizing their work conducted behind the scenes.*”

[2025-Y1-039].

The image is reminiscent of the public displays of gratitude for health care providers early in the COVID-19 pandemic. In this drawing, other professionals are elevated to the status of super-hero (depicted wearing capes), while the pharmacist’s work is not visible.

### 3.5. Health Care Provider

This aspect of identity is linked to pharmacists’ clinical involvement in patient care and as academic clinicians [60]. The white coat symbolizes this aspect of identity. This identity discourse places importance on professional titles and activities typically associated with physicians. Conversely, it tends to downplay the significance of the dispenser and merchandiser aspects of identity [60].

The role of pharmacists as healthcare providers appeared in over 40% of the pSquares. This aspect of identity was indicated by the white coat. Pharmacists were depicted as involved in clinical activities, collaboration with physicians and other health care providers, the administration of vaccinations, and the delivery of other public health services. Their accessibility as primary health care providers was emphasized. Illustrations of medications appeared, which is in keeping with discourse related to the health care provider aspect of identity and is similar to the dispenser discourse.

**Figure 7:**
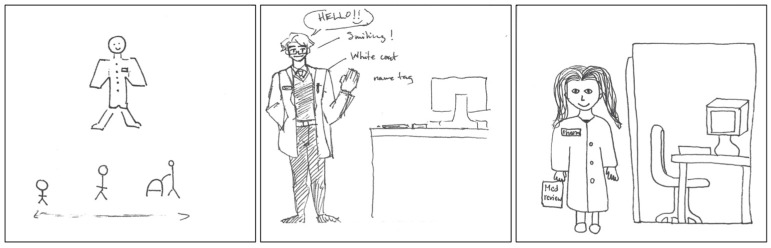
pSquares depicting the white coat symbol of the health care provider aspect of identity (2025-Y1-015, 2025-Y1-045 with text “Hello!! Smiling! White coat. Name tag.”, 2025-Y1-054).

The pSquares in Figure 7 highlight the professional image of pharmacists in white coats, caring for patients of all ages and providing clinical services such as medication reviews.

**Figure 8:**
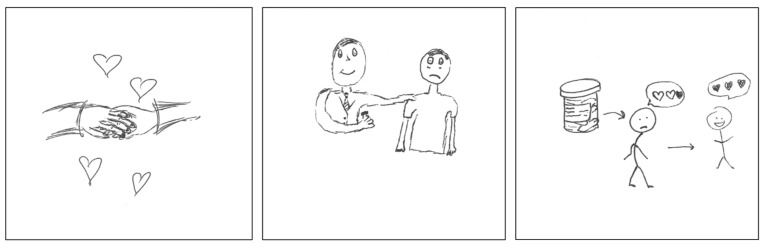
pSquares depicting the caring aspect of the health care provider identity (2025-Y1-005, 2025-Y1-051, 2025-Y1-023).

Clinical aspects of the pharmacist’s role in delivering patient care were represented through visual imagery of pharmacists delivering care, heart symbols, and intimate and close interactions between pharmacists and patients. The third pSquare in Figure 8 emphasized medications, symbolized by a vial, in association with the health care provider aspect of the pharmacist role. In this particular drawing, the pharmacist is actively providing patient care and the result is a favorable patient outcome, as indicated by the presence of hearts and the accompanying text:

“*Pharmacists provide care to patients in need of assistance through medication.*”

[2025-Y1-023].

The pharmacist’s provision of medication demonstrated care and support for the patient.

**Figure 9:**
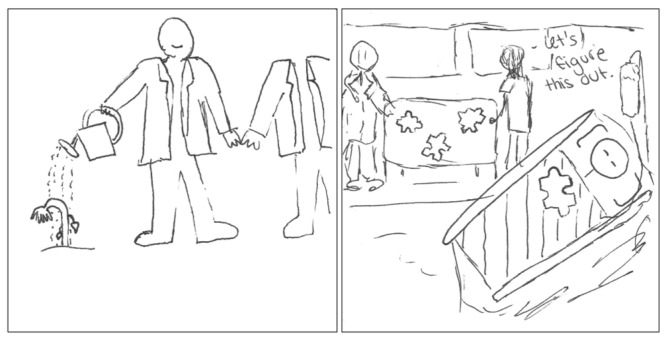
pSquares depicting the collaborator role (2025-Y1-013, 2025-Y1-077 with text “Let’s figure this out.”).

As health care providers, pharmacists were portrayed as equal partners, collaborating with other health care providers such as physicians. In Figure 9, the drawings on the pSquares serve to legitimize the clinical elements of the pharmacist role and their collaborative efforts alongside physicians as health care providers. The accompanying written text is as follows:

“*I’ve drawn a pharmacist watering a wilted plant to represent them helping an ill patient replenish and become healthy. They are also holding hands with another professional to show they aren’t alone. We work together with other healthcare professionals to increase public health.*”

[2025-Y1-013].

“*Every patient has their own puzzle. We need to find your piece.*”

[2025-Y1-077].

**Figure 10:**
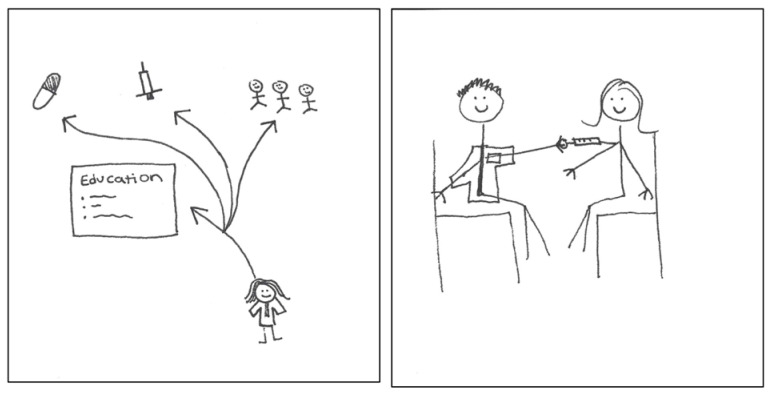
pSquares depicting the public health provider aspect of identity (2025-Y1-008 with text “Education”, 2025-Y1-016).

The health care provider aspect of the pharmacist role also represented other domains of public health. The first pSquare in Figure 10 [2025-Y1-008] depicted pharmacists’ involvement in providing vaccinations and community education outreach. In the second pSquare, the participant recognized the following:

“*Pharmacists play a major role in giving immunizations to help protect our communities.*”

[2025-Y1-016].

**Figure 11:**
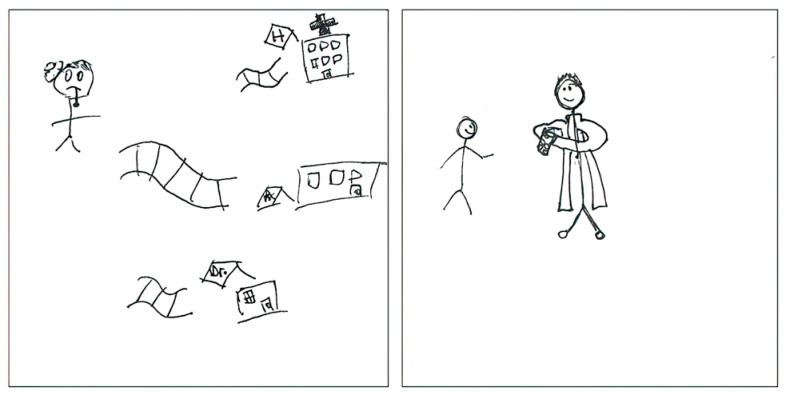
pSquares depicting access to Primary Health Care services (2025-Y1-065 with text “H. Rx. Dr.”, 2025-Y1-064).

Pharmacists were depicted as an important point of contact in primary health care services in the dataset, highlighting an essential aspect of their identity as health care providers. The first pSquare in Figure 11 depicts pharmacists as the initial point of contact for health services, alongside physicians’ offices and hospitals, emphasizing their accessibility. The text accompanying the second image recognizes the scope of practice of pharmacists, including the privilege of prescribing medication. The text reads as follows:

“*I drew a pharmacist prescribing medication to a patient.*”

[2025-Y1-064].

### 3.6. Multi-Faceted Professional

**Figure 12:**
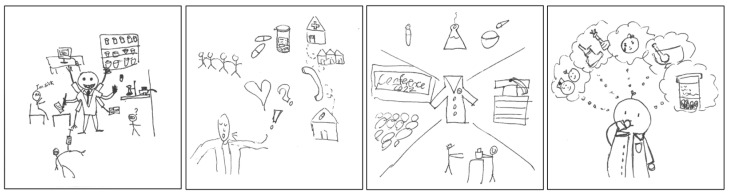
pSquares depicting the multi-faceted professional (2025-Y1-071 with text “I’m sick”, 2025-Y1-074, 2025-Y1-020 with text “Conference 2022”, 2025-Y1-082).

Another discourse, herein labelled the multi-faceted professional discourse, refers to the multiple aspects of identity associated with the pharmacist role. Figure 12 provides examples of pSquares that depict the coexistence of these multiple aspects of identity. The first pSquare portrays an “octopus-like pharmacist” image, suggesting the simultaneous performing of various tasks and potential overlap of roles. Similarly, the fourth pSquare displays a range of activities in thought bubbles, which could be interpreted as either confusing or flexible, depending on the specific patient or situation. The text accompanying the drawings on these pSquares highlighted the diverse and multi-faceted identity of pharmacists in society, also referring to the potential for expansion in the future:

“*Patient care, research, education, drug synthesis, consultation, practice, expansion.*”

[2025-Y1-071].

“*I highlighted the roles of pharmacists in communication (with other healthcare workers, care homes, and neighbourhoods) and general tasks like dispensing and answering questions and empathizing/checking for alarming interactions. Also they advocate and consult patients and for the profession.*”

[2025-Y1-074].

“*In the center, I drew a signature white coat of a pharmacist. Around it I drew lab materials (compounding), books (academia), giving drugs (practice), and a conference (professional development).*”

[2025-Y1-020].

“*I drew a few different roles that popped up when thinking about a pharmacist, such as: dispensing medication, compounding, helping patients, etc.*”

[2025-Y1-082].

## 4. Discussion

This study provides a baseline understanding of perceptions of the pharmacist role among first-year pharmacy students. Using an adapted draw-and-write technique, students expressed their perceptions of the pharmacist role. The results showed that students associated pharmacists with five established identity discourses: apothecary, dispenser, merchandiser, expert advisor, and health care provider [60]. The dispenser and health care provider aspects of the pharmacist role were frequently portrayed in community pharmacy settings, while fewer drawings depicted other aspects of identity. Dispensing encompassed communication, patient counseling, and compounding. Participants’ drawings also depicted activities related to health care teams, public health, and primary health care. Some drawings featured the historical apothecary identity, while others emphasized more modern aspects of the pharmacist role as primary care provider. Other potential role and identity categories [23] were expressed textually including scholar, in which pharmacists are engaged in a range of activities including research, drug discovery, teaching, and knowledge dissemination; health advocate, which entails advocating for patients and the pharmacy profession; and professional, in which pharmacists are continually learning and participating in professional development activities such as conferences. We highlight the coexistence of multiple, concurrent, overlapping, and competing aspects of role and identity captured by the multi-faceted professional discourse.

The findings of our analysis indicate that the student participants from the class of 2025 had a comprehensive understanding of the pharmacist role upon entering the Doctor of Pharmacy program. This result differs from those of previous research. Previous studies have reported limited knowledge of pharmacists’ responsibilities among prospective health care students in the US [38] and fourth-year pharmacy students in Canada [39] and Australia [1]. This difference may be attributed to various factors, such as admission requirements, students’ prior experiences with pharmacists and the profession, and the increased recognition of pharmacists’ contributions during the COVID-19 pandemic [62]. Looking at the demographics of the class of 2025, we see that the participants in this study may have had prior work experience in health care settings or prior employment in pharmacies, which may have influenced their understanding of the pharmacist role. These findings point to the possibility that professional identity formation is already underway when students enter the program.

Participants’ drawings prominently featured symbols of medication, which is consistent with previous research that highlighted the dispensing aspect of pharmacists’ identities [46,63]. However, unlike in previous research, the medication symbols in the drawings in our study were associated with the health care provider aspect of identity. This association of medication symbols with both the dispenser and health care provider aspects of identity requires further investigation. It may simply indicate a recognition of pharmacists’ expertise with medications [23], and refer to pharmacy’s historical roots, or it may emphasize the significance of medications in health care.

In our study, a diverse array of activities were portrayed by pharmacy students in their drawings, reflecting the multi-faceted professional discourse. This discourse acknowledges the dynamic nature of modern pharmacy practice, blending historical identities, like the apothecary and dispenser, with contemporary activities associated with health care providers and other professionals. Previous research has addressed multiple roles and discourses. Elvey and colleagues [64] describe pharmacists embracing multiple identities. Discussing the fluidity of professionals’ roles, Jarvis and Gouthro [65] acknowledge the possibility of multiple identity discourses. Kellar et al. [60] caution against the potential confusion caused by merging identities, aptly called the discursive “pile-up,” and its impact on students’ professional identity formation. The multi-faceted discourse warrants further exploration to better understand its influence on professional identity formation and evolution, as well as the enactment of associated roles in modern pharmacy practice.

In the process of professional identity formation, the arts can disrupt and refocus how professional roles are represented, thereby contributing to the construction of new professional identity discourses [65]. In their review of the literature on arts and professional education, Jarvis and Gouthro [65] assert that engaging students with art reveals multiple perspectives and understandings of professional work. Arts-based approaches foster critical thinking and encourage students to challenge assumptions, allowing them to develop new perspectives and see beyond the traditional norms of their professions. The arts also play a vital role in developing the capacity to tolerate ambiguity and navigate multiple discourses [66]. The arts are therefore invaluable in educational settings as they equip learners with the skills to question, imagine, reimagine, and engage in discussions about the complexities of their future professional roles.

Integrating arts-based activities in pharmacy education can stimulate discussions and reflections on the dynamic nature of the pharmacist role, thus facilitating the formation of professional identity. Educators can use arts-based activities as a starting point for reflective conversations with students and to complement other instructional approaches. Various art forms, such as drawing, photography, and poetry, support reflection and enhance professional identity formation [32,65]. Building on the findings of this study, educators can utilize the draw-and-write technique to explore the range of meanings and discourses represented in students’ drawings. Furthermore, additional elements, such as theory [67], an established curriculum using the arts [34], and models to integrate arts and humanities [68], can further support professional identity formation in the pharmacy curriculum. Pharmacy educators can also employ reflexive techniques to examine their own perceptions of the pharmacist role and aspects of identity, as well as the language and symbols used in teaching, as their representation of various identity discourses will impact their students [60,69].

This study uses a social constructionist approach to explore the pharmacist role and aspects of identity depicted in students’ drawings. This theoretical perspective emphasizes the influence of dominant discourses on the understanding and enactment of professional roles. It is crucial to acknowledge how discourse may shape students’ comprehension and the enactment of professional roles, thus influencing the formation of their professional identities [18]. Furthermore, students’ prior experiences and future socialization with pharmacists may aid in the construction and reframing of their own professional identities and influence how these identities are projected to others [20].

Despite the valuable insights gained from this study, it has certain limitations. Participants were asked to generate a drawing within a limited time frame of 7 min. While this allowed for spontaneous and immediate responses, it also constrained the level of detail and complexity that could be captured in the drawings. It is important to note that drawings alone do not provide enough detail about the intended meaning. As a result, some nuances of students’ perspectives may have been overlooked or not fully represented. In future studies, the drawing time could be extended, or additional activities could be incorporated, to illuminate students’ perceptions further. The utilization of interviews or focus groups would improve our understanding of students’ perceptions and interpretations of the depicted symbols and their relationship to the pharmacist role.

This study’s findings provide several implications for future research. Longitudinal studies tracking students’ conceptions of professional identity throughout their pharmacy education would provide valuable insights into students’ development as professionals and facilitate the evaluation of the impact of the curriculum on their evolving understanding of the pharmacist role. A subsequent study involving the same cohort of students, such as the Class of 2025, could shed light on how their understanding of pharmacists’ roles evolves over time as they progress through the Doctor of Pharmacy program, thus influencing their professional identity formation. Arts-informed research methods could be used to explore the challenges students face as they navigate the ever-changing and diverse health care system, particularly as they work with pharmacists during their clinical placements [26].

In response to calls from pharmacy researchers, educators, and students for a unified collective professional identity [3,70,71,72], this study contributes to the conversation about professional identity and introduces the multi-faceted professional identity discourse, which is characterized by diverse and sometimes conflicting responsibilities. Future research efforts will continue to find ways to support students’ professional identity formation and an understanding of the pharmacist role, helping them to “think, act, and feel” like pharmacists, starting the first day of their program.

## 5. Conclusions

The arts-informed research approach utilized in this study, the draw-and-write technique, provided valuable insights into the perceptions of first-year pharmacy students regarding the pharmacist role. The students’ drawings revealed their understanding of the role, and they placed particular emphasis on the dispenser and health care provider aspects of identity. The multi-faceted professional discourse highlighted the coexistence of multiple aspects of identity within contemporary pharmacy practice. The integration of arts-based activities into the curriculum has the potential to enhance students’ professional identity formation. Future studies utilizing the draw-and-write technique could consider adjustments to address the limitations related to drawing time and may be complemented with other research methods to provide a more comprehensive understanding of students’ perceptions. Longitudinal studies are necessary to delve deeper into students’ perceptions and experiences of the pharmacist roles within pharmacy programs. A more comprehensive understanding of the multi-faceted nature of the pharmacist role may inform the development of educational strategies to support professional identity formation effectively. The findings of this study shed light on the complexities of the pharmacist role and the potential for arts-based methods to support professional identity formation within pharmacy education.

## Figures and Tables

**Table 1:** Participant demographics.

Demographics		Participants (%)
Gender	Female	59
	Male	39
	Non-binary	1
	Prefer not to disclose	1
Age (years)	<20	6
	20–24	82
	25–29	11
	30–34	0
	35–39	0
	>40	1

## Data Availability

The data presented in this study are openly available in ERA: Education and Research Archive, University of Alberta, at https://doi.org/10.7939/r3-xyta-5g55 (accessed on 25 August 2023).
